# Acute exacerbation of major depressive disorder following valbenazine treatment for tardive dyskinesia: A case report

**DOI:** 10.1002/pcn5.70204

**Published:** 2025-09-14

**Authors:** Fumiaki Yano, Yasunori Oda, Yuki Hirose, Fumiaki Yamasaki, Yusuke Nakata, Tomihisa Niitsu

**Affiliations:** ^1^ Department of Psychiatry Chiba University Graduate School of Medicine Chiba Chiba Japan; ^2^ Department of Child Psychiatry Chiba University Hospital Chiba Chiba Japan

**Keywords:** antipsychotics, depression, tardive dyskinesia (TD), valbenazine, vesicular monoamine transporter 2 (VMAT2) inhibitors

## Abstract

**Background:**

Tardive dyskinesia (TD) is a movement disorder associated with long‐term use of dopamine receptor‐blocking agents. Valbenazine, a selective vesicular monoamine transporter 2 (VMAT2) inhibitor, effectively reduces TD symptoms, but it may also trigger or worsen depressive symptoms by reducing central dopamine and serotonin availability.

**Case Presentation:**

We report the case of a 52‐year‐old woman with major depressive disorder (MDD) and oral dyskinesia who experienced an acute mood deterioration after she began taking valbenazine 40 mg/day. She developed TD while receiving antipsychotic treatment for depression. After she achieved partial psychiatric improvement on lurasidone, we started valbenazine to address her dyskinesia. Within hours of the first dose, she reported markedly worse depressive symptoms, heightened anxiety, and significant functional decline, even though her dyskinesia improved. Her Hamilton Depression Rating Scale score increased from 30 before valbenazine to 40 the next day. We stopped valbenazine, and over the following month, she gradually regained mood stability.

**Conclusion:**

This case shows that valbenazine can acutely worsen preexisting depression. Clinicians should actively evaluate psychiatric history, assess current mood stability before prescribing, and monitor patients closely to identify and address mood changes promptly.

## BACKGROUND

Tardive dyskinesia (TD) is a neurological disorder that can result from prolonged exposure to dopamine receptor‐blocking agents, primarily antipsychotic medications. It presents with involuntary, repetitive, and abnormal movements affecting the orofacial region, trunk, and/or limbs. TD can significantly impair patients' quality of life and functional capacity and is often challenging to manage effectively once established.^1^ Management strategies for TD include dose reduction or discontinuation of the offending antipsychotic, switching to another antipsychotic. However, these approaches are not always effective, necessitating the addition of pharmacological interventions.^2^

Vesicular monoamine transporter 2 (VMAT2), such as valbenazine, deutetrabenazine, and tetrabenazine, has demonstrated clinical efficacy for TD.^3^ VMAT2 is a membrane‐associated protein expressed in the central and peripheral nervous systems, which facilitates the transport of monoamines, including dopamine, from the cytoplasm into synaptic vesicles. Inhibition of VMAT2 reduces dopamine release into the synaptic cleft by limiting its vesicular storage.^4^ One proposed pathophysiological mechanism of TD involves chronic blockade of dopamine D2 receptors, which triggers a compensatory increase in D2 receptor density.^5^ As endogenous dopamine levels remain stable, this upregulation increases dopaminergic input to the receptors. By reducing dopamine release into the synaptic cleft, VMAT2 inhibitors may alleviate TD symptoms.

Although VMAT2 inhibitors effectively reduce dopamine release to alleviate TD symptoms, they may also exacerbate depressive symptoms. According to the monoamine hypothesis of depression, major depressive disorder (MDD) is associated with deficient levels of serotonin and dopamine in the central nervous system. Consistent with this, experimental studies have demonstrated that VMAT2 inhibition decreases serotonin^6^ and dopamine^7^ levels, providing a mechanistic rationale for its potential to worsen mood symptoms.

Herein, we report a case of acute exacerbation of MDD following valbenazine administration for TD. Although the patient's oral dyskinesia showed marked improvement, her depressive symptoms significantly worsened, necessitating valbenazine discontinuation. Her psychiatric condition gradually stabilized over approximately 1 month following cessation of the medication. Written informed consent was obtained from the patient for the publication of this case report, including clinical details.

## CASE PRESENTATION

The patient was born at full term without perinatal complications and achieved normal developmental milestones. After graduating from high school, she was employed as a print checker until age 24, when she left her job upon marriage. She subsequently became a homemaker and is the mother of one son. Her past medical and psychiatric histories were unremarkable. In March 2020, at age 48, she presented to a general hospital with a persistent low‐grade fever. Despite concerns regarding influenza or SARS‐CoV‐2 infection, no specific abnormalities were identified, and she was managed with observation. She became increasingly anxious due to her inability to obtain polymerase chain reaction testing for COVID‐19 and began reporting altered taste perception. She developed persecutory ideation, believing she would be mistreated if she sought further medical attention. In April 2020, following a local COVID‐19 outbreak linked to a nearby sushi restaurant, she developed the delusion, despite family reassurance, that she was responsible for the outbreak. This event appeared to precipitate the onset of depressive symptoms, including low mood, anhedonia, excessive guilt, and recurrent thoughts of death. In May 2020, accompanied by her family, she consulted a psychiatric clinic. She was diagnosed with MDD with mood‐congruent psychotic features, and olanzapine 5 mg/day was initiated. According to the outside‐clinic referral, olanzapine monotherapy appeared to have been chosen to rapidly control the prominent mood‐congruent delusions. Her condition initially improved; however, a subsequent dose reduction due to significant weight gain led to a symptomatic relapse. Aripiprazole 3 mg/day was added, but her mood did not improve, and she was referred to our hospital in March 2023. At referral, her medication regimen consisted of olanzapine 15 mg/day, aripiprazole 3 mg/day, and duloxetine 20 mg/day. At that time, according to the Diagnostic and statistical manual of mental disorders, fifth edition,^8^ she met criteria for a current major depressive episode. The symptom constellation included depressed mood, markedly diminished interest/pleasure, insomnia, psychomotor agitation, diminished energy, excessive guilt, and recurrent thoughts of death, occurring on most days for more than 2 weeks with associated functional impairment. Because her inability to remain still and the constant need to move were suggestive of akathisia, olanzapine was tapered to 10 mg/day, and aripiprazole was discontinued. The restlessness subsequently improved; however, depressive symptoms persisted, and lurasidone 20 mg/day was initiated in November 2023, after which her psychiatric condition gradually improved. In December 2023, she developed involuntary tongue movements, including protrusion, consistent with oral dyskinesia. At that time, the Abnormal Involuntary Movement Scale (AIMS) total score^9^ was 4 (lips/perioral area, 2; tongue, 2). On January 31, 2024, despite an improved mood and preserved insight, her oral dyskinesia worsened, and the AIMS total score increased to 6 (lips/perioral area, 3; tongue, 3). Because lurasidone had been associated with a clinically meaningful improvement in depressive and anxiety symptoms, we judged that the overall risk–benefit balance favored continuing lurasidone rather than stopping it immediately. Therefore, with plans for close monitoring and reassessment of antipsychotic need, valbenazine 40 mg/day was initiated. That same night, she reported a marked recurrence of depressive symptoms, including depressed mood, diminished motivation, and anxiety with agitation. On the other hand, her oral dyskinesia resolved abruptly within a few days of valbenazine treatment; tongue protrusion disappeared, and the AIMS total score decreased to 1 (lips/perioral area, 1). Nevertheless, within 1 week, she presented for an unscheduled visit due to a significant deterioration in daily functioning: she was no longer able to cook, became easily irritable, and her depressive symptoms worsened markedly. During this time, suicidal ideation emerged and was assessed by clinical interview. A safety plan was instituted consisting of weekly outpatient follow‐up over the next week, and the patient agreed to contact our hospital immediately in the event of escalating risk, with inpatient admission to be considered. Given the clear temporal association with valbenazine initiation, the drug was discontinued. Following valbenazine cessation, her psychiatric symptoms gradually improved over approximately 1 month. Her Hamilton Depression Rating Scale (HAM‐D) scores were 29 at her initial visit to our hospital, 30 before lurasidone initiation, 40 on the day following valbenazine initiation, and 27 one month after its discontinuation.

To address alternative explanations: no new psychosocial stressors were identified; physical examination was unremarkable; and laboratory studies, including thyroid function tests, were normal. Akathisia had resolved after tapering olanzapine and discontinuing aripiprazole and was absent at the time of mood worsening, with no psychotropic dose changes after initiation of lurasidone 20 mg/day; together, these findings support a temporal link between valbenazine initiation and the depressive exacerbation.

## DISCUSSION

We report the case of a patient with MDD who took valbenazine for TD. Although her oral dyskinesia was alleviated dramatically, she experienced an acute exacerbation of depressive symptoms following the initiation of valbenazine. Whereas valbenazine has demonstrated efficacy and safety in multiple clinical trials,^10^, ^11^, ^12^, ^13^, ^14^ our findings underscore the need for vigilance in clinical practice regarding the potential emergence or worsening of depressive symptoms.

The clinical trials often excluded individuals with active mood symptoms, with representative studies setting the cutoff at a Montgomery–Åsberg Depression Rating Scale (MADRS) score of approximately >13, thereby excluding those with more than mild depressive symptoms. This raises questions about the generalizability of such findings to real‐world populations with comorbid depression. Furthermore, in the KINECT 4 trial, a 52‐week open‐label study, suicidal ideation led to treatment discontinuation in three participants, and in one case (a 66‐year‐old male), the investigator judged the adverse event as possibly related to the study drug.^13^ In addition, a published case report by Neeman et al. describes a 49‐year‐old man with schizoaffective disorder who developed significant depression and suicidal ideation on Day 4 of valbenazine treatment for TD.^15^ His symptoms returned to baseline by Day 6 after valbenazine discontinuation. Although his underlying diagnosis differed from that of our patient, the acute onset of depressive symptom exacerbation after valbenazine initiation and the subsequent improvement following discontinuation closely parallel the clinical course we observed.

Other VMAT2 inhibitors—such as tetrabenazine, which has been associated with depression and suicidal ideation in multiple clinical trials,^16^ and deutetrabenazine, for which a real‐world safety analysis reported similar findings^17^—have been linked to these adverse effects. Pharmacologically, valbenazine is a highly selective VMAT2 inhibitor with minimal activity at VMAT1 and other off‐target sites.^18^ Compared with tetrabenazine and deutetrabenazine, these differences in selectivity, pharmacokinetics, and the extent of central monoaminergic depletion may partly account for the observed differences in psychiatric safety profiles. However, given that all VMAT2 inhibitors share the core mechanism of reducing presynaptic dopamine and other monoamines, the potential for depressive symptom emergence or worsening remains biologically plausible in susceptible individuals, as illustrated by our case.

Accordingly, these observations underscore the need for caution when prescribing valbenazine to patients with a history of mood disorders. Thorough baseline psychiatric assessment and close monitoring are essential to detect early signs of mood destabilization and mitigate the potential risk of psychiatric deterioration.

## CONCLUSION

This case report highlights a rare but clinically significant adverse reaction to valbenazine in a patient with comorbid MDD and TD. The temporal association between drug initiation and psychiatric worsening underscores the need for vigilance among clinicians when prescribing valbenazine to individuals with preexisting or unstable affective symptoms. Regular psychiatric monitoring and individualized treatment planning are paramount to ensuring both the safety and efficacy of TD management in this vulnerable population.

## AUTHOR CONTRIBUTIONS

Fumiaki Yano and Yasunori Oda drafted the manuscript and reviewed the patient's clinical information for this report. All authors were involved in the clinical management of the patient and have read and approved the final manuscript.

## CONFLICT OF INTEREST STATEMENT

The authors declare no conflicts of interest.

## ETHICS APPROVAL STATEMENT

N/A.

## PATIENT CONSENT STATEMENT

N/A.

## CLINICAL TRIAL REGISTRATION

N/A.

## Data Availability

Data sharing is not applicable to this article as no datasets were generated or analyzed during the current study.
